# Apoptosis of Hepatocytes: Relevance for HIV-Infected Patients under Treatment

**DOI:** 10.3390/cells10020410

**Published:** 2021-02-16

**Authors:** Aleksandra Gruevska, Ángela B. Moragrega, Andrea Cossarizza, Juan V. Esplugues, Ana Blas-García, Nadezda Apostolova

**Affiliations:** 1 Department of Pharmacology, Faculty of Medicine, University of Valencia, 46010 Valencia, Spain; agruevs@alumni.uv.es (A.G.); anbemoes@alumni.uv.es (Á.B.M.); nadezda.apostolova@uv.es (N.A.); 2 FISABIO—Hospital Universitario Dr. Peset, 46017 Valencia, Spain; ana.blas@uv.es; 3 Department of Medical and Surgical Sciences for Children and Adults, University of Modena and Reggio Emilia, 41124 Modena, Italy; andrea.cossarizza@unimore.it; 4 National Institute for Cardiovascular Research, 40126 Bologna, Italy; 5 National Network of Biomedical Research on Hepatic and Digestive Diseases (CIBERehd), 46010 Valencia, Spain; 6 Department of Physiology, Faculty of Medicine, University of Valencia, 46010 Valencia, Spain

**Keywords:** antiretroviral drugs, HIV, liver, toxicity, apoptosis, hepatic cell death

## Abstract

Due to medical advances over the past few decades, human immunodeficiency virus (HIV) infection, once a devastatingly mortal pandemic, has become a manageable chronic condition. However, available antiretroviral treatments (cART) cannot fully restore immune health and, consequently, a number of inflammation-associated and/or immunodeficiency complications have manifested themselves in treated HIV-infected patients. Among these chronic, non-AIDS (acquired immune deficiency syndrome)-related conditions, liver disease is one of the deadliest, proving to be fatal for 15–17% of these individuals. Aside from the presence of liver-related comorbidities, including metabolic disturbances and co-infections, HIV itself and the adverse effects of cART are the main factors that contribute to hepatic cell injury, inflammation, and fibrosis. Among the molecular mechanisms that are activated in the liver during HIV infection, apoptotic cell death of hepatocytes stands out as a key pathogenic player. In this review, we will discuss the evidence and potential mechanisms involved in the apoptosis of hepatocytes induced by HIV, HIV-encoded proteins, or cART. Some antiretroviral drugs, especially the older generation, can induce apoptosis of hepatic cells, which occurs through a variety of mechanisms, such as mitochondrial dysfunction, increased production of reactive oxygen species (ROS), and induction of endoplasmic reticulum (ER) stress and unfolded protein response (UPR), all of which ultimately lead to caspase activation and cell death.

## 1. Introduction

The effectiveness of the anti-human immunodeficiency virus (HIV) treatments currently available has reduced the likelihood of acquired immune deficiency syndrome (AIDS)-defining illnesses and prolonged the life expectancy of HIV patients. This is resulting in non-AIDS conditions becoming more common in these individuals [1], of which liver-related diseases are increasingly prominent [2]. Among the molecular mechanisms activated in the liver during HIV infection, apoptotic cell death of hepatocytes stands out as a key pathogenic player. In this review, we will describe the most important mechanisms of apoptosis reported in hepatocytes in vitro and in animal models induced by HIV, its viral proteins, or the currently used anti-HIV drugs (excluding the eight drugs already discontinued by the Food and Drug Administration (FDA)).

### 1.1. HIV: Infection and Therapy

Recent medical and sociological advances have dramatically improved the progression of HIV infection worldwide. Nevertheless, current statistics of the global HIV/AIDS burden reflect what continues to be an important pandemic, with 38 million infected, of whom 25.4 million are in treatment [3]. The number of HIV cases continues to rise, particularly in vulnerable populations and under-resourced areas; it has risen by 21–72% since 2010 depending on the region. In 2019, 690,000 lives were lost due to AIDS-related diseases, and approximately 1.7 million people were infected with HIV [3]. On a more positive note, there is a trend towards fewer new HIV infections; since 2010, they have diminished by 23%, thanks in large part to a major decrease (38%) in eastern and southern Africa [3].

Infection with HIV, a lentivirus [4], is possible due to interaction of the envelope (Env) glycoproteins present on the surface of the virus (i.e., gp120 and gp41) with, respectively, the receptor CD4 and the chemokine co-receptors (CXCR4 and CCR5) located on the primary target cells (activated CD4^+^ T cells). The virus can also infect other cell types, such as resting CD4^+^ T cells, macrophages, monocytes, and dendritic cells [5,6]. Additionally, infection can occur independently of the CD4 receptor, as reported for astrocytes [7] and renal tubular epithelial cells [8]. Inside the CD4^+^ T cell, the virus releases RNA and enzymes, and uses reverse transcriptase (RT) to convert its genetic material—HIV RNA—into DNA, which enters the nucleus and combines with the host cell’s DNA through the activity of viral integrase.

The different steps of the viral life cycle within the host cells can be inhibited by several commercially available molecules. So far, 30 antiretroviral (ARV) drugs and a monoclonal antibody (ibalizumab) have been approved for clinical use, and they are categorized in the following drug families: nucleoside and nucleotide reverse transcriptase inhibitors (NRTIs), non-nucleoside reverse transcriptase inhibitors (NNRTIs), protease inhibitors (PIs), integrase strand transfer inhibitors (INSTIs), HIV entry inhibitors (CCR5 antagonists), HIV fusion inhibitors, and attachment and post-attachment inhibitors (as shown in Table 1). ARV regimens, referred to as combined antiretroviral therapy (cART), generally consist of a combination of drugs of the different families (in some cases, together with a pharmacokinetic enhancer or booster, of which cobicistat and ritonavir are the most frequently chosen options) [9]. cART was designed to limit the probability of drug resistance, to reduce specific drug side effects by diminishing their individual dosages, and to obtain a synergic effect between drugs that act on different molecular targets [10].

Lack of drug adherence is one of the main problems of anti-HIV treatments. An important strategy to improve drug adherence is to use fixed-dose combinations (FDCs) which include multiple classes of ART in one tablet taken once daily, known as a single tablet regimen (STR). FDCs can be co-administered with another ARV agent to create a multiple-tablet regimen that can be taken once or more times per day, depending on its components. Currently, there are 23 FDCs approved by the FDA, of which 19 are STRs. While the potential of the individual drugs that compose STRs to induce hepatocyte apoptosis has been addressed (mainly in vitro), there is a lack of information about the effect of ARV combinations or STRs.

Other limitations of cART include drug resistance and safety issues. In this regard, toxicity and adverse effects have been described with all ARV drugs, are often enhanced due to drug-drug interactions, and are the most common reason for discontinuing therapy. Overall, gastrointestinal disturbances, hypersensitivity and skin reactions, neuropsychiatric disorders, and liver toxicity tend to appear early after initiation of treatment, while other side effects, such as lipid and glucose metabolism abnormalities, kidney alterations, bone metabolic disorders, and mitochondrial toxicity generally develop after chronic exposure [11].

Due to the fact that cART achieves extended virologic suppression of HIV replication but does not eradicate it, patients are exposed to ARV compounds for decades, which can result in accumulated toxicity, one of the three factors that can contribute to a greater risk of chronic diseases in these patients. The other two are i) HIV itself and the effects of chronic viral infection and associated chronic, persistent inflammation; and ii) the host, most notably the population and psychosocial context in which the HIV disease occurs. For example, rates of hepatitis B and C infection, obesity, and tobacco use, all of which raise a patient’s risk of suffering a variety of chronic illnesses, are highly prevalent among HIV-infected patients, often at rates that substantially exceed those in the general population [12]. Hence, treatment guidelines recommend regimens based not only on their antiviral potency, but also on their chronic toxicity.

Hepatotoxicity, or drug-induced liver injury (DILI), is one of the most common side effects of cART, and is responsible for morbidity, mortality, and discontinuation of ARV treatment [13,14]. In addition, liver injury is thought to be HIV-induced—a hypothesis supported by many clinical studies—and there is a special emphasis on the association between HIV RNA content and liver fibrosis development [15,16].

Severe liver toxicity can be defined as the presence of an increase in plasma aspartate transaminase (AST), alanine transaminase (ALT), and alkaline phosphatase levels over five-fold the normal upper limit [17]. Following this criterion, many studies have shown that increased liver transaminase levels occur in 2–18% of patients after several months on cART [18], while a rate of up to 30% has been reported in others [19,20]. HIV infection itself elevates liver transaminase levels, and in some cases liver injury may be exacerbated when HIV patients undergo treatment [21,22]. However, it is difficult to ascertain liver toxicity associated with each ARV drug, because they are administered in combination; moreover, patients are also exposed to other liver toxins and/or often have preexisting hepatic conditions. In addition to this, other risk factors have been associated with liver injury induced in HIV-infected patients.

Another important non-drug-related factor is chronic hepatitis produced either by hepatitis C (HCV) or hepatitis B (HBV) virus, as co-infected patients display greater increases in plasma levels of liver enzymes compared to HIV mono-infected individuals. Other factors, like severe alcohol consumption, drug abuse, drug interactions, age, gender, or ethnicity also contribute to liver injury [23]. The severity of this hepatic injury ranges from transient rises in liver enzyme levels to more severe clinical events, such as hepatitis, non-alcoholic fatty liver disease (NAFLD), or steatohepatitis (NASH), non-cirrhotic portal hypertension or even acute liver failure, which can lead to death [13]. Importantly, liver-related mortality ranks as second cause of AIDS-unrelated death in HIV-infected patients [24].

### 1.2. HIV Pathogenesis and the Liver

The liver is a central organ involved in a wide range of essential functions of the organism, such as metabolism, glycogen storage, drug detoxification, production of serum proteins, and bile secretion. For these purposes, it requires a high level of vascularization, slow blood flow, and a highly permeable fenestrated endothelium. There are two major liver cell populations: parenchymal cells (or hepatocytes), which comprise 80% of total liver cells, and non-parenchymal cells, including liver sinusoidal endothelial cells (LSECs), hepatic stellate cells (HSCs), hepatic dendritic cells (DCs), and Kupffer cells (KCs), the liver’s resident form of macrophages [25]. This review will specifically focus on the death of hepatocytes, given that it is a common denominator of virtually all types of liver injury and the basis for inflammation/fibrosis promotion.

HIV may alter liver function by direct or indirect mechanisms. It is arguable whether HIV is able or not to infect hepatocytes, as they are not considered to be a target of the virus, even though viral particles have been found in this organ [26]. However, HIV RNA has been detected in primary human hepatocytes obtained from liver biopsies of infected patients [27] or transfected with the virus in vitro [28,29]. On one hand, it has been reported that the Huh7.5 hepatoma cell line is slightly susceptible to HIV infection because of inefficient fusion, which is due to an absence of cell surface CD4 and CXCR4 receptors [30]. In this context, plasma membrane glycosphingolipids, such as glycolipid galactosyl ceramide, have been proposed as an alternative entry point for HIV into hepatocytes [30]. On the other hand, various studies have confirmed that productive infection of HIV in primary hepatocytes and hepatoma cell lines is CD4-independent [31,32]. The level of HIV infection in hepatocytes in vitro is low, but it can be significantly potentiated by “second hits”, including co-infection or alcohol [28,29]. Specifically, the ethanol metabolite acetaldehyde was reported to increase HIV RNA levels in primary human hepatocytes and Huh7.5 cells [29]. HIV can also infect different liver cell types other than hepatocytes. It has been shown to infect KCs and may disrupt their number or function in HIV-infected patients [33,34]. An in vitro study showed that primary HIV isolates can also infect both LX-2, an HSC line, and primary human HSCs [35]. In addition, viral antigens, part of infectious virions, or defective virions unable to productively infect any cell type, may affect liver cell populations without the need for viral infection [33]. In the case of HIV, these soluble antigens consist largely of gp120 and the trans-activator protein Tat [12], whose potential proapoptotic mechanisms in hepatocytes are discussed below.

### 1.3. Apoptosis and the Liver

Apoptosis, the best understood form of programmed cell death, is a strictly regulated process that takes place as a response to either receptor-mediated (Fas, TNFR1, DR3, DR4, and DR5 receptors) or non-receptor-mediated (such as DNA damage) signals [36,37]. Apoptosis has a role in many normal physiological processes, including homeostasis of lymphocyte populations, tissue differentiation, and elimination of tumorigenic, mutated, or virus-infected cells. The response to death signals varies depending on the cell type, activation or developmental stage of the cell, and the environment. Two major apoptotic pathways have been described: the extrinsic, which originates at the death receptors on the cell membrane, and the intrinsic, in which mitochondria are major players [38]. Importantly, certain stimuli can activate both pathways and are interconnected, since the process of apoptosis converges with the activation of a series of cysteine-aspartic proteases known as caspases, categorized in three major groups: initiators, executioners, and inflammatory caspases. Upon cell damage, initiator caspases (caspases 2, 8, 9, and 10) are activated (from inactive procaspases) and in turn activate the executioner caspases (caspases 3, 6, and 7), which initiates a cascade of events culminating in DNA fragmentation from activation of endonucleases, destruction of nuclear proteins and the cytoskeleton, crosslinking of proteins, ligand expression for phagocytic cells, and the formation of apoptotic bodies [38]. A second group of proteins, the Bcl-2 family, regulates the apoptotic pathway. These proteins contain anti-apoptotic (Bcl-2, Bcl-XL, BAG) and pro-apoptotic (Bax, Bak, Bid) members that exert their function inside mitochondria, either by compromising them or inducing their dysfunction. The anti-apoptotic proteins localize to the outer mitochondrial membrane, whereas the pro-apoptotic ones are sequestered in the cytosol. Upon receiving a death signal, the pro-apoptotic proteins translocate to the mitochondrion to interact with anti-apoptotic counterparts, thereby promoting mitochondrial dysfunction. Finally, activation of caspase 3 represents the point of no return and results in the activation of downstream effectors, leading to the typical apoptotic features described above [39].

Although apoptotic cell death can occur in all cell types in the liver, it is that of hepatocytes which has been studied in greatest detail and is considered the basis of liver damage in many different circumstances. Factors that trigger apoptotic liver injury can be classified into three groups: external factors, including hepatotropic viruses, and xenobiotics, such as alcohol and drugs; internal factors (endobiotics), derived from the liver itself, for example, toxic bile acids and free fatty acids (FFAs); and immune factors (immune-mediated mechanisms), which share similarities with both the internal and the external factors. Apoptosis of hepatocytes is a fundamental component of virtually all liver diseases, as the ensuing responses of cell repair, regeneration, inflammation, and fibrosis can all be triggered by apoptosis. In fact, an increase in the plasma levels of several hepatic enzymes (mainly transaminases), an effect established as a proxy for liver damage, occurs as a result of hepatic cell death. The engulfing of apoptotic bodies originating from dying hepatocytes by HSCs or KCs and other macrophages stimulates fibrogenesis and inflammation, thus further promoting liver disease. In support of the role of hepatocyte apoptosis in advanced liver pathogenesis, it is relevant that cytokeratin-18 (CK-18), a marker of hepatocyte apoptosis, which occurs in NASH but not in NAFLD, is the most validated diagnostic biomarker of NASH. Moreover, hepatocyte injury and increased turnover are also essential for the development of liver cell cancer.

There is clinical evidence of the importance of hepatocyte apoptosis in the development of liver disease in HIV patients. In this sense, a recent study has shown the correlation between high plasma levels of hyaluronic acid, a relevant liver fibrosis marker, and caspase-cleaved CK-18 in patients with HIV/HCV co-infection [40]. Similarly, biopsies from HCV/HIV co-infected patients show that higher number of hepatocytes express the death receptor Fas (CD95, a member of a subgroup of the tumor necrosis factor (TNF) receptor superfamily) and undergo irreversible apoptosis compared to biopsies from HCV-infected patients [41]. Moreover, the proportion of Fas-immunoreactive hepatocytes was associated with the stage of liver fibrosis. Similarly, a higher degree of intrahepatic apoptosis was detected in liver biopsies from patients co-infected with HIV and HBV than in those from HBV monoinfected patients. Based on morphology, the majority (75%) of the TUNEL-positive (apoptotic) cells were identified as hepatocytes [42].

## 2. HIV, HIV-Encoded Proteins, and Apoptosis

### 2.1. HIV as an Inducer of Hepatocyte Apoptosis

A study with a humanized mouse model of chimeric mice dually reconstituted with a human immune system and HIV-infected hepatocytes reported various mechanisms by which HIV contributes to hepatocyte apoptosis. HIV-infected animals showed a decline in human albumin (ALB) concentration and a significantly reduced percentage of human hepatocytes, as well as a decline in CD4^+^ T cells in the liver and an increase in peripheral HIV viral load [43] compared to uninfected mice. Serum ALB level is characterized as a useful marker of the progression of HIV-associated liver disease [44]. Moreover, when liver immune activation was evaluated, a pro-inflammatory response that appeared to contribute to the depletion and dysfunction of hepatocytes was observed. Finally, the study confirmed the ability of HIV to trigger hepatocyte apoptosis in vivo [43]. Another investigation with chronically HIV-infected humanized mice also revealed a reduction of ALB levels and restoration of liver synthetic function with long-acting nanoformulated ARV drugs (atazanavir and ritonavir) [45].

HIV-induced apoptosis in hepatocytes is potentiated by ethanol, as its main metabolite acetaldehyde enhances accumulation of HIV components (HIV-gag RNA and p24) in hepatocytes due to their impaired degradation by the lysosome and the proteasome. When combined with acetaldehyde, these HIV components induce oxidative stress, abortive HIV infection of hepatocytes, and apoptotic cell death, as shown by one in vitro study [29].

### 2.2. HIV Proteins and Apoptosis

HIV infection is well known to be associated with a progressive decline in CD4^+^ T cell number, which is the cause of immunodeficiency and susceptibility to opportunistic pathogens and malignancies. Many studies have demonstrated that, during HIV infection, both infected and uninfected T cells undergo apoptosis [46,47,48], and six of the eight HIV-encoded proteins—Env (both gp120 and gp41), Tat, Nef, Vpr, Vpu, and protease (PR)—have been shown to induce this form of programmed cell death. Three of them—Env and the regulatory proteins Tat and Nef—share the capacity to induce cell-surface expression of death ligands and receptors of the TNF family [49], whereas PR-induced apoptosis is associated with the loss of mitochondrial integrity [50].

In addition to HIV infection of hepatocytes, it has been suggested that hepatocytes exposed to HIV are injured through an “innocent bystander” mechanism after cell-surface binding of viral proteins.

#### 2.2.1. gp120-Mediated Apoptosis

**Figure 1:**
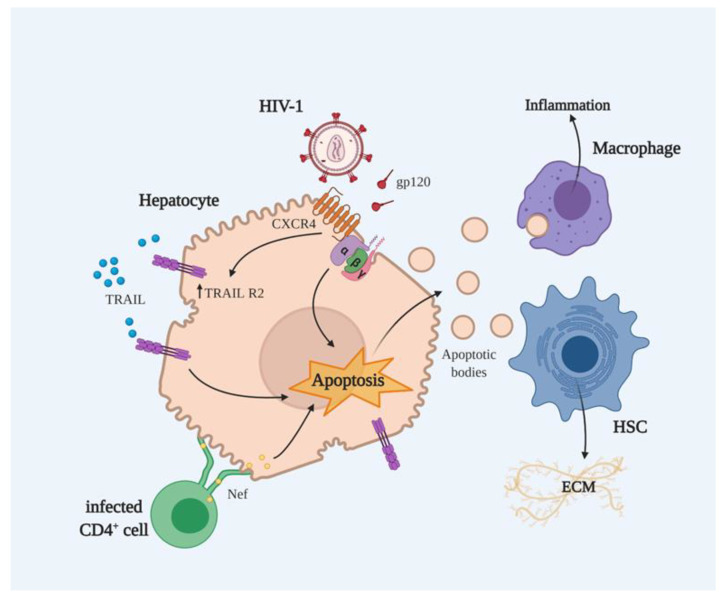
Possible mechanisms of the involvement of human immunodeficiency virus (HIV)-encoded proteins Env and Nef in the apoptosis of hepatocytes and the development of liver fibrosis, as suggested mainly by in vitro observations. The binding of gp120 to CXCR4 induces hepatocyte apoptosis in a Giα protein–dependent manner. CXCR4 signaling also up-regulates TRAIL R2 sensitizing to TRAIL-induced death. TRAIL levels are increased during HIV infection and this effect contributes to the ability of gp120 to induce apoptosis. For its part, Nef is able to transfer from CD4^+^-infected cells to hepatocytes through conduits, thereby contributing to the induction of apoptosis. The resulting apoptotic bodies are engulfed by hepatic stellate cells (HSCs), an effect by which quiescent HSCs are converted into activated HSCs, the main profibrogenic cell type whose ability to secrete extracellular matrix (ECM), among other actions, is fundamental for the development of liver fibrosis.

The HIV protein Env is initially synthesized as the large precursor molecule gp160, which is then cleaved into secreted gp120 and the transmembrane gp40. Gp120 cross-linking of CD4 induces susceptibility to Fas-mediated killing. In activated cells, gp120 cross-linking also results in apoptosis (possibly mediated by IFN-γ, TNF, or both) [51], down-regulates Bcl-2 expression [52] and mediates activation of caspase 3 [53]. Gp120 can also induce cell death in uninfected CD4^+^ T cells as a part of circulating immune complexes and replication-incompetent viruses, since it does not require viable virions to exert these effects [49]. Moreover, gp120 binding to CD4 may activate CD95/CD95L-dependent or, alternatively, Bax-dependent, intrinsic apoptotic pathways [54]. Interactions between gp120 and CXCR4 can cause mitochondrial membrane permeabilization (MMP) through pertussis toxin-sensitive G proteins (Giα), the p38 MAPK pathway, and/or Ca^2+^-dependent mechanisms [55]. Some studies have demonstrated HIV’s ability to directly cause the death of hepatocytes, regardless of whether or not they are infected. The presence of CXCR4 in hepatocytes allows them to interact with gp120. Thus, gp120 binds to CXCR4, inducing hepatocyte apoptosis without infecting the cell, even in the absence of the whole virus [56]. Unlike gp120/CXCR4-mediated CD4^+^ T lymphocyte death, available data indicate that gp120/CXCR4-mediated hepatocyte death is signaled through the Giα protein coupled to the CXCR4 receptor and is independent of the caspase cascade [56] (Figure 1). Moreover, there is a second mechanism through which CXCR4 induces apoptosis in the surviving hepatocytes, as CXCR4 signaling also up-regulates TRAIL R2 expression and provides an acquired sensitivity to TRAIL-induced death (Figure 1). This fact is especially relevant considering that TRAIL levels in plasma are elevated during HIV infection [57] and in other liver diseases, like HBV or HCV infection, thereby accelerating liver disease. Indeed, an in vitro study with Huh7.5.1 cells showed that hepatocyte apoptosis is increased in the presence of HCV and HIV compared to HCV or HIV alone, and that this increase is mediated by DR4 (TRAIL R1) and DR5 (TRAIL R2) up-regulation [58]. Otherwise, it has been reported that the phagocytic clearance of apoptotic bodies resulting from HIV-infected and HCV/HIV co-infected hepatocytes can also lead to activation of the inflammasome in macrophages and of HSC activation, therefore contributing to the progression of liver disease [28,29,59] (Figure 1). These in vitro studies using hepatocytes seeded on polydimethyl siloxane (PDMS) gels—thereby mimicking a fibrotic liver environment—also demonstrated that increased liver stiffness potentiates apoptotic cell death in co-infected hepatocytes, thus accelerating the progression of liver disease in HCV/HIV+ patients with established liver fibrosis [28].

#### 2.2.2. Nef-Mediated Apoptosis

The HIV protein Nef is crucial for viral pathogenicity and has been proposed as a mediator of apoptosis due to its ability to induce the expression of pro-apoptotic molecules by activating the Fas/Fas ligand signaling pathway in CD4^+^ lymphocytes [60]. Some studies show that this protein plays an essential role in the development of different HIV-related diseases, mainly because of its capacity to migrate from HIV-infected to HIV-uninfected bystander CD4^+^ T cells, and even to B cells, which are generally resistant to the virus. This is due to its ability to attack the host cell membrane and rearrange the cytoskeletal structure and organelle formation, as well as to alter the immunological synapse, effects that have a bearing on the protein-trafficking process [61,62]. One study that employed a co-culture of Jurkat T cells and a hepatic cell line reported that Nef was transferred from Jurkat T cells to hepatocytes through conduits [63]. Many of the known functions of Nef are relevant to the process of intercellular transmission; hence, it is reasonable to assume that Nef expressed by HIV infected T cells, macrophage/monocytes, and/or dendritic cells travels to hepatocytes through conduits and alters the course of HCV-mediated liver disease [63] (Figure 1). Finally, both Nef and HCV core proteins activate TNF receptor-associated factors (TRAFs), which play a central role in the regulation of many biological activities, such as immune and inflammatory responses, and apoptosis [64].

## 3. cART-Related Apoptosis in Hepatocytes

### 3.1. NRTIs

Generally, the molecular mechanisms of NRTI-driven liver injury involve alterations in cell morphology, mitochondrial integrity, and endoplasmic reticulum (ER) function, which can lead to altered mitochondrial bioenergetics and toxicity, hypersensitivity reactions, and hepatic steatosis [65]. Below, we describe their effects regarding apoptosis in the liver. For NRTIs to be effective, they need to be taken up by the host cell, phosphorylated to the triphosphate form, bind at the polymerase active site, mimic a substrate that can be incorporated into viral DNA by HIV RT, and, finally, act as chain terminators. The main toxicity of these drugs is due to inhibition of DNA polymerase γ, an enzyme required for mitochondrial DNA (mtDNA) replication [66]. mtDNA encodes 13 subunits of the electron transport chain (ETC) that are essential to oxidative phosphorylation. Therefore, in addition to apoptosis, NRTI-induced depletion of mtDNA leads to enhanced mitochondrial ROS production and an impairment of fatty acid oxidation and microvesicular hepatic steatosis [67].

**Figure 2:**
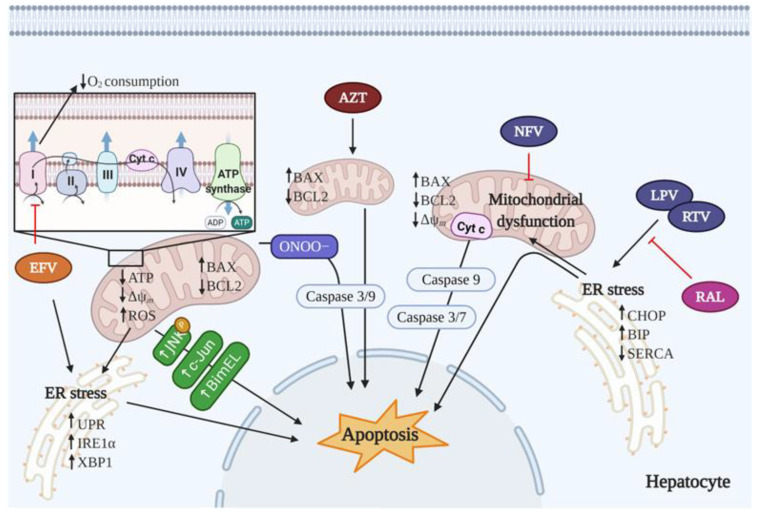
Potential molecular mechanisms through which antiretroviral (ARV) drugs can induce apoptosis of hepatocytes, as suggested mainly by in vitro studies. Efavirenz (EFV) disrupts mitochondrial function and produces apoptosis by direct inhibition of complex I activity of the mitochondrial electron transport chain (ETC), leading to a decrease in total oxygen consumption, an increase in production of reactive oxygen species (ROS) and a decrease in Δψ_m_. EFV triggers endoplasmic reticulum (ER) stress and activates unfolded protein response (UPR) to finally produce apoptosis. In addition, EFV mediates cell death through c-Jun N-terminal kinase (JNK) and Bcl-2 interacting mediator of cell death (Bim) activation. AZT increases the expression of Bax and diminishes the expression of Bcl-2, which leads to caspase 3 activation and apoptosis. RTV and LPV also induce ER stress and UPR, effects that can derive in mitochondrial dysfunction, which can trigger apoptosis. In contrast, the anti-apoptotic action of NFV occurs through the preservation of mitochondrial Δψ_m_ and the inhibition of subsequent post-mitochondrial signaling events, such as activation of caspase 3. RAL prevents LPV/RTV-induced apoptosis by inhibiting ER stress and caspase 9 activation. AZT, zidovudine; EFV, efavirenz; NFV, nelfinavir; LPV, lopinavir; RTV, ritonavir; RAL, raltegravir.

Zidovudine (3′-azido-3′-dexoythymidine, AZT) was the first anti-HIV agent, approved in 1987. A decade later, a combination with lamivudine was registered, followed by a combination with lamivudine and abacavir. Structurally, AZT is an analogue of thymidine in which the 3′-hydroxyl group is replaced by an azido group. An in vitro study reported a significant concentration-dependent decrease in the number of viable cells in the human hepatoma cell line HepG2 and immortalized human liver cell line THLE2 following 4-week AZT treatment and a 1-week recovery period, with much higher concentrations being required to induce cell death in the latter cell line. The decrease in cell viability was attributed to a combination of effects, including cell cycle arrest, induction of apoptosis, and a decrease in telomerase activity [68]. The same research group demonstrated incremented AZT-DNA incorporation, diminished cell growth, enhanced apoptotic and necrotic cell death, and increased cell cycle arrest in AZT-treated cells in which XPC, a protein responsible for recognition of distorted DNA structure, had been knocked down. These results suggest that XPC, a key factor of the nucleotide excision repair (NER) pathway, is crucial for DNA damage recognition and repair after AZT treatment in human hepatoma cells [69]. Other in vitro experiments also demonstrated that AZT inhibited HepG2 cell growth and induced intracellular lipid accumulation without affecting mtDNA [70]. AZT was found to suppress both constitutive and induced autophagy in human hepatocytes, which resulted in increased ROS content and triggered apoptosis [71]. Autophagy is an intracellular process of lysosomal degradation and recycling of proteins and organelles. It is a major pathway for mitochondrial turnover, which targets dysfunctional mitochondria for breakdown, and inhibition of autophagy per se has been associated with the accumulation of dysfunctional mitochondria [72]. Furthermore, the ability of AZT to inhibit autophagy correlates with its potential to induce intracellular lipid accumulation in hepatocytes, which could contribute to liver injury, particularly in diseases in which additional metabolic, infectious (HCV or HBV) or drug-related toxicity can accumulate. Lastly, several in vivo studies with rats and mice treated with AZT illustrated the pro-apoptotic effects of this NRTI. In AZT-treated rats, a significant increase in the expression of the pro-apoptotic protein Bax and a marked decrease in the expression of the anti-apoptotic protein Bcl-2 was produced within the liver [73] (Figure 2). Similarly, livers from AZT-treated B6C3F1 mice showed increased expression of pro-apoptotic genes (Bax, Bbc3, and Bnip3) [74]. It is noteworthy that, in both in vivo studies, a supraclinical dose of AZT was employed. It is because of this high toxicity and severe adverse effects that the use of this drug is currently very limited. Aside from AZT, other NRTIs have been reported to alter cell proliferation in HepG2 cells. At C_max_ concentrations, emtricitabine (FTC) induced moderate effects, and carbovir, the active form of abacavir, considerably impaired proliferation in this cell line. In both cases, NRTI-induced effects were independent of mtDNA depletion. Didanosine reduced HepG2 cell growth while decreasing mtDNA levels, but only at supratherapeutic concentrations [75]. However, these actions have not been related to the induction of apoptotic pathways.

### 3.2. NNRTIs

NNRTIs bind to a hydrophobic pocket distal to the active site within the RT, causing a stereochemical change in the RT protein, which reduces the ability of naturally occurring nucleosides to bind to the active site pocket, thus impeding viral DNA synthesis [76].

Nevirapine (NVP), the first approved NNRTI (1996), has a low genetic barrier to resistance, low clinical potency, and can cause serious adverse effects (e.g., life-threatening hypersensitivity reaction). With respect to liver toxicity, NVP inhibits proliferation of both HepG2 and THLE2 cells to a similar degree, leading to necrotic cell death [77]. Nevertheless, the concentrations that cause these actions were shown to be higher than average plasma concentrations. The highest concentration or prolonged treatment with this NNRTI were reported to produce G2/M and G1/G0 phase cell cycle arrest in HepG2 and THLE2 cells, respectively. Further experiments indicated that NVP induced cellular senescence in THLE2 cells, but not in hepatoma cells, while there was evidence of a lack of apoptosis in both cell lines [77]. NVP is metabolized by cytochrome P450, resulting in the formation of four mono-oxygenated metabolites, of which 12-hydroxy-NVP (12-OHNVP) is associated with hepatotoxicity and skin toxicity [78,79]. In a recent study using primary mouse hepatocytes, supra-therapeutic concentrations of NVP-induced cell death, while a version of the drug in which the twelfth-position had been trideuterated (12-D3NVP) exerted a lesser effect in comparison to NVP [80]. In contrast, in another study assessing the toxicity of two-day exposure of primary hepatocytes from five human donors to NVP or ritonavir (RTV), the former inhibited EIF2 signaling and was predicted to suppress downstream ATF4- and CHOP-related apoptotic pathways (revealed by analysis of transcriptomic data) and PDI and heat shock responses. Interestingly, these effects were more evident with the supra-therapeutic concentration [81].

Efavirenz (EFV) became commercially available in 1998, and in the following two decades was widely employed as part of several successful therapeutic regimens administered as first-line treatment. With the arrival of newer ARV drugs (like INSTIs) with a better toxicological profile and similar efficacy, EFV lost its prominence within initial cART regimens. Because of its numerous side effects, including hepatic alterations, EFV is no longer recommended as a part of first-choice cART. Various in vitro studies reviewed in [82], many of them performed in hepatocytes, indicate that short-term treatment with clinically relevant concentrations of EFV reduces cellular proliferation and/or compromises cell viability. Our group reported that treatment of Hep3B and primary cultures of human hepatocytes with EFV resulted in specific inhibition of complex I of the ETC, leading to reduced O_2_ consumption, decreased mitochondrial membrane potential (ΔΨ_m_), bioenergetic and metabolic changes, and elevated ROS generation [83,84,85]. These alterations were paralleled by reduced cellular proliferation and the triggering of apoptosis. Furthermore, in line with these manifestations, EFV triggered ER stress and activated the unfolded protein response (UPR) [86]. Excessive or prolonged ER stress can ultimately induce apoptosis, an outcome documented in several human pathologies, including liver disease [87] (Figure 2). Similarly, another in vitro study showed that incubation of primary human hepatocytes with synthetic 8-hydroxyEFV (8-OHEFV), the primary metabolite of EFV, resulted in cell death, caspase 3 activation and ROS formation [88]. It was highlighted that this metabolite is a more potent stimulator of cell death than EFV, and that both EFV and 8-OHEFV mediate cell death through c-Jun N-terminal kinase (JNK) and Bcl-2 interacting mediator of cell death (Bim) (Figure 2). JNKs are a family of serine/threonine kinases that are important regulators of cellular responses to stress, including modulation of cell death. A recent study by the same group demonstrated that EFV and several EFV-like compounds activate key cell stress controllers such as inositol-requiring 1α (IRE1α) and X-box binding protein 1 (XBP1), a regulator of the mammalian UPR [89]. The molecular mechanism by which EFV activated IRE1α and, subsequently, IRE1α-catalyzed splicing of XBP1 mRNA involved increased formation of ROS, an event that can lead to activation of the ER stress response, including the IRE1α-XBP1 signaling axis, whose activation was demonstrated in humans, mice, and macaques. Thus, activation of the IRE1α-XBP1 axis is thought to contribute to EFV-mediated hepatocyte death [89] (Figure 2). In another investigation, this NNRTI was used as a model of drug-induced inhibition of complex I in primary mouse hepatocytes, and the authors concluded that this effect was the cause of cytotoxicity, as inhibition of complex I resulted in increased oxidative stress via the formation of peroxynitrite, which can trigger mitochondria-mediated cell death [90]. Studies performed in our laboratory have demonstrated that the effects of EFV are drug-specific, as clinically relevant plasma concentrations of other ARV drugs (rilpivirine (RPV), raltegravir (RAL), and darunavir (DRV)) did not affect mitochondrial function or undermine cell viability of cultured hepatocytes [91].

### 3.3. PIs

Protease inhibitors (PIs) interfere with the last step of the viral replication cycle by targeting the aspartyl protease of HIV, thus inhibiting the cleavage of viral polyproteins and the subsequent generation of individual viral proteins. RTV is currently used as a pharmacokinetic enhancer of other PIs due to its ability to inhibit the cytochrome P450 3A4. Hepatotoxicity is a significant adverse event of PI-containing cART. The potential to induce hepatotoxicity differs among commercially available PIs and can be enhanced by co-infection with hepatitis viruses, intravenous drug use, and alcohol [92]. The ability to induce ER stress is a common molecular mechanism described for PI-induced side effects such as dyslipidemia, insulin resistance (IR), and lipodystrophy/lipoatrophy, and has been related to the pathogenesis of different types of human liver diseases, including NAFLD and DILI [93]. ER-stress and UPR have been implicated in processes that initiate apoptosis in liver disease [94].

A comparative study using several human cell lines, including HepG2 and peripheral blood mononuclear cells (PBMCs), characterized lopinavir (LPV) as the most potent ER stress inducer among nine FDA-approved PIs (SQV, saquinavir; RTV, ritonavir; IDV, indinavir; NFV, nelfinavir; APV, amprenavir; LPV; TPV, tipranavir; ATV, atazanavir; DRV), followed by RTV and SQV, whereas IDV, TPV, and DRV had no effect regarding ER stress. In line with these results, LPV-injected mice showed higher expression of CHOP in the liver with respect to controls, while RTV produced a slight increase and DRV did not alter CHOP expression [95]. CHOP, a major transcription factor involved in ER stress-mediated apoptosis, has been described as a key player in RTV and LPV-induced apoptosis in murine primary hepatocytes and *Ddit3*^−/−^ mice [96].

Some studies with different human hepatocyte cell lines (L-02, HepaRG and Huh-7.5) have shown that RTV exerts an anti-proliferative effect [97,98]. Moreover, it can induce apoptosis in hepatocytes via the caspase-cascade system by enhancing caspase 3 cleavage [97]. The mechanism suggested for RTV-induced cytotoxicity linked ER stress with mitochondria-mediated apoptosis [98]; namely, in Huh-7.5 cells, treatment with RTV enhanced the expression of the ER stress mediator BiP, linked in turn to Bax relocation at the mitochondrial outer membrane, which caused MMP. Concurrently, the drug’s entry into mitochondria diminished ΔΨ_m_. Both outer and inner mitochondrial membrane damage lead to a release of cytochrome *c*, which executes mitochondria-mediated apoptosis by activating caspase 9; this activates caspase 7, which performs cleavage of PARP-1 (Figure 2) [98]. In another study, rats treated with RTV exhibited hepatotoxicity due to increased transaminases and oxidative stress, with a reduction of GSH levels and enhanced ROS and NO production [99]. In addition, RTV displayed an inflammatory effect, causing an increase in the levels of the inflammatory cytokines TNF-α, IL-1β, and IL-6, and a decrease in the anti-inflammatory mediator IL-10. RTV’s apoptotic effects were corroborated by increased protein expression of Bax, caspase 3, and caspase 8, and diminished levels of Bcl-2 [99]. A study using the hepatic cell lines HepG2 and WCH-17 cells showed that NFV promoted apoptotic cell death and induced cell cycle arrest in G0/G1 [100].

As previously mentioned, a considerable proportion of HIV-infected patients concomitantly consume alcohol, which can not only impair patients’ adherence to cART but also exacerbates ARV drug-induced hepatotoxicity, thus leading to greater morbidity and mortality [101,102]. In mouse and human primary hepatocytes, LPV/RTV treatment combined with alcohol potentiated the ER stress induced by these PIs. The combination of alcohol with these HIV drugs also inhibited sarcoplasmic reticulum Ca^2+^-ATPase (SERCA) expression, which regulates the calcium store and ER homeostasis, thus promoting cell death [103]. Another study confirmed enhanced cell death produced in hepatocytes in response to a combination of alcohol and LPV/RTV [104]. In addition, the study in question reported that treatment with LPV/RTV inhibited Nrf2, a transcription factor that regulates the expression of many genes required for antioxidant defenses [104]. In line with these results, RTV and ATV, but not APV, induced ER stress and activated UPR in primary rat hepatocytes [105]. In a transcriptomic study using an in vitro flow-based culture system with human primary hepatocytes that resembles liver-derived hemodynamic blood flow parameters, RTV was predicted to activate UPR and downstream pathways in a concentration-dependent manner, thus leading to apoptosis. Moreover, RTV increased the activity of complexes I and IV, with simultaneous uncoupling and inhibition of complex V. These circumstances could contribute to mitochondrial dysregulation, ultimately leading to necrosis and apoptosis of hepatocytes [81].

In contrast to the previously described pro-apoptotic effects, some PIs have been shown to be anti-apoptotic. A study evaluated the impact of NFV, boosted with RTV, on hepatocytes isolated from mice that were challenged ex vivo with Jo-2 (an anti-Fas antibody) in order to induce excessive apoptosis, whilst mice were pretreated with NFV/RTV. This pretreatment did not alter caspase 8, but significantly inhibited caspase 3 activity. Additionally, NFV/RTV pretreatment reversed the loss of ΔΨ_m_ observed in control hepatocytes. Therefore, it was suggested that NFV’s anti-apoptotic action was possible through the preservation of mitochondrial ΔΨ_m_ and subsequent postmitochondrial signaling events [106] (Figure 2).

### 3.4. INSTIs

INSTIs inhibit HIV by blocking the strand transfer step of viral DNA integration into the host genome. However, few studies have focused on the capacity of INSTIs to induce apoptosis in liver cells. RAL was the first INSTI approved for clinical use (2007). Experiments performed in primary rat hepatocytes and livers of mice treated with RAL, with or without RTV and LPV, revealed that RAL did not induce cell apoptosis, and even prevented it by inhibiting ER stress and LPV/RTV-induced caspase 9 activation [107] (Figure 2). As previously mentioned, studies performed by our group have demonstrated that RAL does not undermine the viability of cultured hepatocytes [91].

### 3.5. Entry and Fusion Inhibitors

Fusion inhibitors were designed to prevent entry of HIV into the host cell by binding to the gp41 protein located in the viral envelope. The binding prevents conformational changes required for fusion of the viral envelope with the cell membrane. To date, enfuvirtide (T-20) is the only fusion inhibitor available [108] and has not been associated with elevations of serum aminotransferase levels [109]. No instances of clinically apparent liver injury due to T-20 are reported in the published literature.

Entry inhibitors, or CCR5 co-receptor antagonists, bind to the hydrophobic pocket within the transmembrane helices of the CCR5 receptor, and they induce and stabilize a receptor conformation, preventing the virus’s entry into the host cell [110]. Maraviroc (MVC), the only CCR5 inhibitor available to treat HIV infection, is active only against HIV strains which have tropism for this receptor, and not for CXCR4 or mixed tropism (CCR5/CXCR4) [111]. One study reported that MVC reduced mortality, liver fibrosis, apoptosis, and tumorigenesis in a mouse model of hepatocellular carcinoma and protected liver cells from free radical-induced cell death [112].

### 3.6. Attachment and Post-Attachment Inhibitors

In 2020, the FDA approved fostemsavir, a prodrug of the first-in-class HIV attachment inhibitor temsavir, which binds directly to the gp120 subunit, thus locking it into a conformational state that prevents its binding with the CD4 T cell receptor, which impedes attachment. Adverse events, such as hepatobiliary abnormalities, appeared in the Phase III clinical trial and were associated with the multiple therapies that patients had been exposed to (heavily-treatment experienced HIV) and the significant presence of medical comorbidities in the study population [113].

In 2018, the FDA approved ibalizumab, the first monoclonal antibody to CD4, which is used to treat patients with multidrug-resistant HIV infection. This biologic drug does not block the binding of gp120 to CD4, but instead inhibits the conformational changes in the gp120/CD4 complex that allow binding to a second cellular receptor, CXCR4, thus inhibiting HIV fusion and entry. Ibalizumab therapy has not been associated with serum enzyme elevations or clinically apparent DILI [114].

## 4. Metabolic Alterations and Hepatic Cell Death

Lipotoxicity is among the main factors contributing to the death of hepatocytes. It primarily results from increased FFAs, the peroxidation of which leads to increased ROS production, mitochondrial dysfunction, and ER stress. This causes metabolic dysregulation, which contributes to the development of various features of the metabolic syndrome, such as IR, dyslipidemia, obesity, and NAFLD, including its more severe form, NASH. Consistent with FFA-mediated hepatocyte apoptosis as a pivotal pathogenic mechanism in NASH, human NASH is characterized by increased hepatocyte apoptosis [77], and serum biomarkers of apoptosis are useful for identifying NASH in humans. NAFLD, considered the hepatic manifestation of the metabolic syndrome, is more prevalent in HIV patients than in the uninfected population, reaching 30–40% [115]. Intra-liver accumulation of FFA is not only a result of altered lipid profile, but also of disturbances of glucose metabolism such as IR, whose prevalence is also increased in HIV patients due to the ongoing immune activation in these individuals [116]. Insulin normally suppresses the activity of hormone-sensitive lipase (HSL), an enzyme present in adipocytes which hydrolyses stored triglycerides into FFAs [117]. In the case of IR, the suppression of HSL is diminished, resulting in increased hydrolysis in peripheral adipose tissue and, in turn, increased delivery of FFAs to the liver [118].

In HIV patients, a number of factors may be responsible for increased hepatic FFA accumulation, including effects induced by ARV drugs, as ARV belonging to different families (but particularly PIs and NRTIs) have been related to IR, dyslipidemia, and lipodystrophy [119]. Multiple mechanisms have been proposed for the ARV drug-mediated increase in FFAs in the liver, including enhanced activity of the nuclear localization of SREBP-1 (sterol regulatory element-binding protein 1), a transcription factor that regulates the expression of genes associated with lipid synthesis, by some PIs [120]; alterations in glucose metabolism (such as impaired cellular glucose uptake due to inhibition of glucose transporters, also attributed to members of the PI family) [121]; and mitochondrial dysfunction, as in the case of early-generation NRTIs [122].

## 5. Conclusions

In an era of successful treatment of HIV infection, people living with the virus continue to face multi-morbidity, with chronic liver disease being among the most prevalent and deadliest non-AIDS-defining chronic conditions. Liver disease is also closely related to the metabolic disturbances these individuals suffer. The mechanisms by which liver injury occurs in HIV patients are numerous, but many converge in a common cellular outcome, i.e., the apoptosis of hepatocytes. In addition to the direct effects exerted by the virus itself or HIV-encoded proteins, apoptosis of hepatocytes is clearly aggravated in these patients by other well-known hepatotoxic insults, such as co-infection or the consumption of alcohol, and by antiretroviral therapy itself. Greater understanding of the individual role of each of these factors in apoptotic pathways is key to improving existing cART and to developing new improved therapeutic strategies for the management of HIV patients with ongoing liver disease. A variety of mechanisms have been reported to induce apoptosis of hepatic cells in the context of HIV, including mitochondrial dysfunction accompanied with altered expression (location) of anti- and pro-apoptotic proteins, increased ROS production, or induction of ER stress and UPR upregulation, all of which ultimately lead to caspase activation. Considering that the burden of NAFLD and NASH is rapidly increasing worldwide, a thorough comprehension of the mechanisms that drive the development of liver disease in people living with HIV is essential to the clinical management of these patients.

## Figures and Tables

**Table 1:** Pharmacological groups, their mechanism of action and the names of the Food and Drug Administration (FDA)-approved antiretroviral drugs. Drugs no longer available or recommended for use are not included.

Drug Class	Mechanism of Action	Drugs
**Fusion Inhibitors**	Interfere with the entry of HIV into cells by inhibiting fusion of viral and cellular membranes	Enfuvirtide
**Entry Inhibitors**	CCR5 antagonist, prevents the interaction with HIV gp120 and prevents the virus from entering the cell	Maraviroc
**Attachment Inhibitors**	Bind to the gp120 protein on the outer surface of HIV, preventing HIV from entering CD4 cells	Fostemsavir
**Post-Attachment Inhibitors**	Inhibit the conformational changes in the CD4/gp120 complex that allow binding to CXCR4, thus inhibiting HIV fusion and entry	Ibalizumab
**Reverse Transcriptase** **Inhibitors** Nucleoside analogues (NRTIs) Nonnucleoside analogues (NNRTIs)	Inhibit the HIV replication by binding directly to the reverse transcriptase (RT) enzyme	Zidovudine Lamivudine Emtricitabine Abacavir Tenofovir Nevirapine Efavirenz Doravirine Etravirine Rilpivirine
**Integrase Strand Transfer** **Inhibitors (INSTIs)**	Block the strand transfer step of viral DNA integration into the host genome	Raltegravir Elvitegravir Dolutegravir Bictegravir
**Protease Inhibitors (PIs)**	Inhibit HIV protease, thereby preventing cleavage of viral proteins and the subsequent generation of individual viral proteins	Ritonavir Saquinavir Lopinavir Atazanavir Fosamprenavir Tipranavir Darunavir
